# Correction: Stoll et al. Impact of HLA-B27 and Disease Status on the Gut Microbiome of the Offspring of Ankylosing Spondylitis Patients. *Children* 2022, *9*, 569

**DOI:** 10.3390/children9081158

**Published:** 2022-08-02

**Authors:** Matthew L. Stoll, Kimberly DeQuattro, Zhixiu Li, Henna Sawhney, Pamela F. Weiss, Peter A. Nigrovic, Tracey B. Wright, Kenneth Schikler, Barbara Edelheit, Casey D. Morrow, John D. Reveille, Matthew A. Brown, Lianne S. Gensler

**Affiliations:** 1 Department of Pediatrics, University of Alabama at Birmingham (UAB), Birmingham, AL 35233, USA; 2 Department of Medicine, Division of Rheumatology, University of Pennsylvania, Philadelphia, PA 19104, USA; kimberly.dequattro@pennmedicine.upenn.edu; 3 Centre for Genomics and Personalized Health, Queensland University of Technology (QUT), Brisbane, QLD 4000, Australia; zhixiu.li@qut.edu.au; 4 Faculty of Health, School of Biomedical Sciences, Queensland University of Technology (QUT), Brisbane, QLD 4000, Australia; 5 Division of Global Migration and Quarantine, Center for Disease Control, Washington, DC 30329, USA; henna.sawhney@gmail.com; 6 Department of Pediatrics and Epidemiology, Perelman School of Medicine, University of Pennsylvania, Philadelphia, PA 19104, USA; weisspa@chop.edu; 7 Division of Rheumatology, Children’s Hospital of Philadelphia, Philadelphia, PA 19104, USA; 8 Division of Rheumatology, Inflammation and Immunity, Brigham and Women’s Hospital, Boston, MA 02115, USA; peter.nigrovic@childrens.harvard.edu; 9 Division of Immunology, Boston Children’s Hospital, Boston, MA 02115, USA; 10 Department of Pediatrics, University of Texas at Southwestern, Dallas, TX 75390, USA; tracey.wright@utsouthwestern.edu; 11 Department of Pediatrics, University of Louisville, Louisville, KY 40292, USA; kenneth.schikler@louisville.edu; 12 Department of Pediatrics, Connecticut Children’s Medical Center, Hartford, CT 06106, USA; bedelhe@connecticutchildrens.org; 13 Department of Cell, Developmental and Integrative Biology, University of Alabama at Birmingham, Birmingham, AL 35294, USA; caseym@uab.edu; 14 Department of Internal Medicine, University of Texas at Houston, Houston, TX 77030, USA; john.d.reveille@uth.thc.edu; 15 Genomics England, London EC1M 6BQ, UK; matt.brown@kcl.ac.uk; 16 Guy’s and St Thomas’ NIHR Biomedical Research Centre, King’s College, London SE1 7EH, UK; 17 Department of Medicine, Division of Rheumatology, University of California at San Francisco, San Francisco, CA 94143, USA; lianne.gensler@ucsf.edu

## Missing Supplementary Materials

The authors wish to make the following correction to this paper [1]. In the original publication, the **Supplementary Materials** were not included. The authors apologize for any inconvenience caused and state that the scientific conclusions are unaffected. The original publication has also been updated.

**Supplementary Materials:** The following supporting information can be downloaded at: https://www.mdpi.com/article/10.3390/children9040569/s1, Table S1: differences between HLA-B27 positive and HLA-B27 negative twins; Table S2: differences between HLA-B27 positive twins and juvenile spondyloarthritis subjects.

## References

[1] Stoll M.L., DeQuattro K., Li Z., Sawhney H., Weiss P.F., Nigrovic P.A., Wright T.B., Schikler K., Edelheit B., Morrow C.D. (2022). Impact of HLA-B27 and Disease Status on the Gut Microbiome of the Offspring of Ankylosing Spondylitis Patients. Children.

